# β‐Thalassemia pathogenic variants in a cohort of children from the East African coast

**DOI:** 10.1002/mgg3.1294

**Published:** 2020-05-11

**Authors:** Alexander W. Macharia, George Mochamah, Sophie Uyoga, Carolyne M. Ndila, Gideon Nyutu, Metrine Tendwa, Emily Nyatichi, Johnstone Makale, Russell E. Ware, Thomas N. Williams

**Affiliations:** ^1^ KEMRI-Wellcome Trust Research Programme Kilifi Kenya; ^2^ Cincinnati Children’s Hospital Medical Center Cincinnati OH USA; ^3^ Department of Medicine Imperial College St Mary’s Hospital London UK

**Keywords:** hematology, hemoglobinopathy, Kenya, Thalassemia

## Abstract

**Background:**

β‐Thalassemia is rare in sub‐Saharan Africa. Previous studies have suggested that it is limited to specific parts of West Africa. Based on hemoglobin A_2_ (HbA_2_) concentrations measured by HPLC, we recently speculated that β‐thalassemia might also be present on the East African coast of Kenya. Here, we follow this up using molecular methods.

**Methods:**

We used raised hemoglobin A_2_ (HbA_2_) values (> 4.0% of total Hb) to target all HbAA members of a cohort study in Kilifi, Kenya, for *HBB* sequencing for β‐thalassemia (*n* = 99) together with a sample of HbAA subjects with lower HbA_2_ levels. Because HbA_2_ values are artifactually raised in subjects carrying sickle hemoglobin (HbS) we sequenced all participants with an HPLC pattern showing HbS without HbA (*n* = 116) and a sample with a pattern showing both HbA and HbS.

**Results:**

Overall, we identified 83 carriers of four separate β‐thalassemia pathogenic variants: three β^0^‐thalassemia [CD22 (GAA→TAA), initiation codon (ATG→ACG), and IVS1‐3ʹ end del 25bp] and one β^+^‐thalassemia pathogenic variants (IVS‐I‐110 (G→A)). We estimated the minimum allele frequency of all variants combined within the study population at 0.3%.

**Conclusions:**

β‐Thalassemia is present in Kilifi, Kenya, an observation that has implications for the diagnosis and clinical care of children from the East Africa region.

## Graphical abstract

Historically, it has generally believed that β‐thalassemia does not occur widely in Africa. The only populations where this condition is known to be present are in Liberia and Nigeria in West Africa. In the current paper we show that β‐thalassemia is present on the East African Coast of Kenya where we have described four separate genetic mutations that together affect approximately 0.3% of the population.

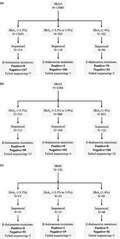

## 1 BACKGROUND

β‐Thalassemia is a heterogeneous group of genetic disorder caused by pathogenic variants in *HBB* that lead to the reduced (β⁺) or absent(β^0^) synthesis of β‐globin (Weatherall & Clegg, 2002). They are examples of balanced polymorphisms, selection having been driven through a survival advantage against *Plasmodium falciparum* malaria in heterozygotes at the expense of the early mortality of homozygotes from intractable anemia (Williams & Weatherall, 2012). Such selection has resulted in current carrier frequencies of 1%–20% in a number of populations including the Mediterranean, the Middle East, India, Southern China, and parts of the Far East (Galanello & Origa, 2010; De Sanctis et al., 2017; Weatherall & Clegg, 2002). In contrast to other red blood cell disorders that have been selected in a similar way, β‐thalassemia is believed to be generally rare within most of sub‐Saharan Africa. Although carrier frequencies of 0.8%–1.7% have been reported in limited parts of Nigeria (Esan, 1970) and Ghana (Weatherall & Clegg, 2002; Weatherall et al., 1971), higher frequencies (of up to 9%) are limited to specific ethno‐linguistic groups within Liberia (Willcox, 1975) and occurrences within East and Central Africa have been largely limited to case reports (McGann et al., 2018).

Recently, we reported that an unexpected proportion of children who we recruited into the REACH trial (NCT01966731), through which we are investigating the use of hydroxyurea among children with sickle cell disease in four African countries, were compound heterozygotes for HbS and β^0^‐thalassemia (HbS/β^0^‐thalassemia;) McGann et al., 2018). Seven percent of participants from our site in Kilifi, Kenya, were carriers of two different β^0^‐thalassemia pathogenic variants. Separately, we have also reported that a small proportion of unselected children recruited to a genetics cohort in Kilifi had suggestive evidence for heterozygous β^0^‐thalassemia based on HPLC‐derived raised HbA_2_ values (Macharia et al., 2019). Here, we have investigated this observation further, through sequencing studies in a subset of children from the latter study.

## 2 RESULTS

**Figure 1:**
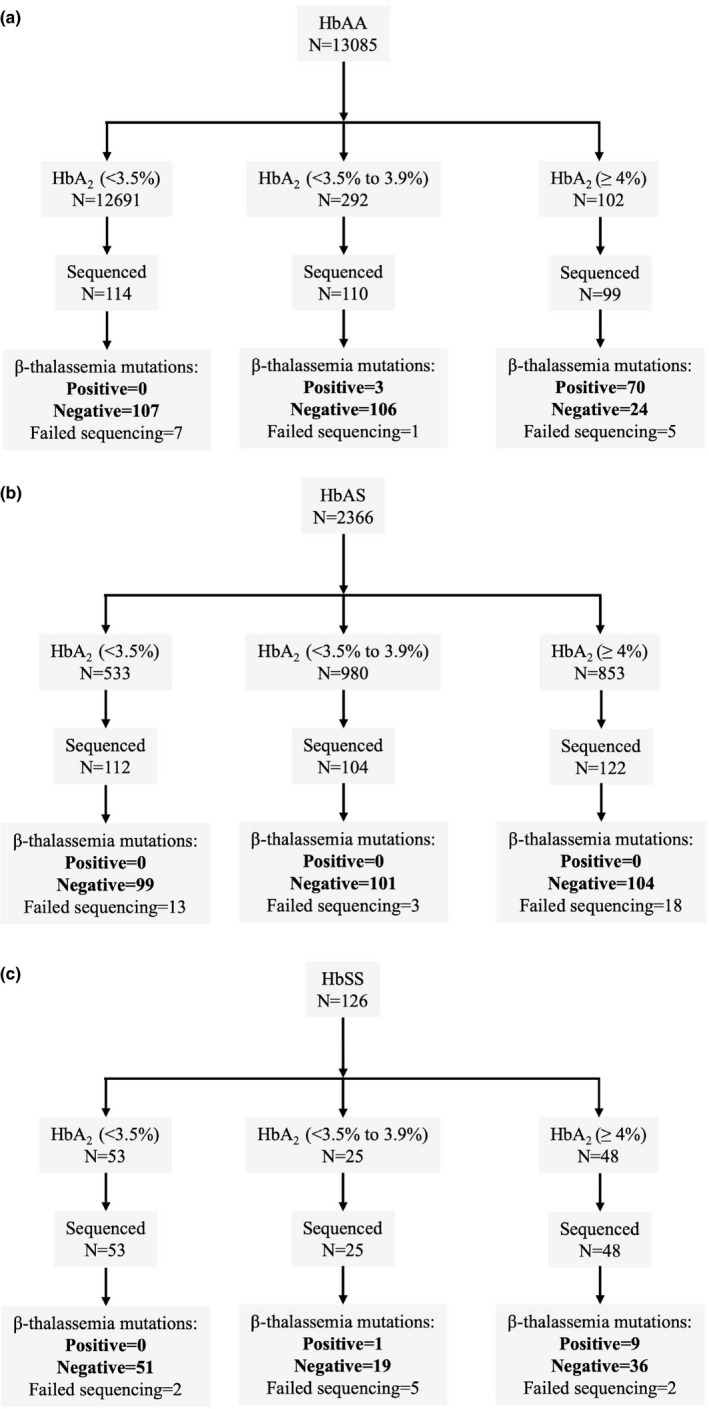
Sample selection and sequencing results for identification of β‐thalassemia pathogenic variants in participants with different HbS phenotypes. *Note*: Total number of study participants were 15,577 distributed as (a) HbAA = 13,085 (84.0%); (b) HbAS = 2,366 (15.2%), and (c) HbSS = 126 (0.8%)

Overall, 15,577 participants were recruited into the Kilifi Genetic Birth Cohort Study at a median age of 6.5 (IQR 5.0–8.4) months. In total, 13,085 (84%) showed an HPLC pattern suggestive of HbAA, 2,366 (15.2%) of HbAS, and 126 (0.8%) of HbSS. On the basis of our sampling strategy, we attempted *HBB* sequencing on 323 participants with an HPLC pattern suggestive of HbAA whose HbA_2_ values were as follows: (1) < 3.5% (*n* = 114); (2) 3.5%–3.9% (*n* = 110); and (3) ≥ 4.0% (*n* = 99; Figure 1a). We also attempted sequencing on 338 participants with an HPLC pattern suggestive of HbAS whose HbA_2_ values were as follows: (1) <3.5% (*n* = 112); (2) 3.5%–3.9% (*n* = 104); and (3) ≥4.0% (*n* = 122; Figure 1b). Finally, we sequenced all children with HbSS (*n* = 126; Figure 1c). Sequencing was successful in 730 of 787 (93%) participants, in whom we identified 83 participants with β‐thalassemia pathogenic variants, all as heterozygous carriers. Four different β‐thalassemia pathogenic variants were observed in total: three β^0^[CD22 (GAA→TAA), a nonsense variant that results in premature termination of β mRNA translation resulting in nonfunctional mRNA (Thein, 2018), initiation codon (ATG→ACG), which abrogates the transfer RNA binding site (Wildmann et al., 1993), and IVS1‐3ʹ end del 25bp], a deletion that results in an inactivation of an acceptor splice site (Orkin, Sexton, Goff, Kazazian, & JR., 1983), along with one β^+^pathogenic variant (IVS‐I‐110 (G→A)). β^0^‐Thalassemia pathogenic variants were the most common, representing 97.6% of all pathogenic variants identified, while CD22 (GAA→TAA) was the most common overall (66.3%; Table 1). Although the group sizes were too small to allow definitive conclusions, we saw no differences in HbA_2_ values between these pathogenic variants (Table 1, Figure S2). One participant with HbS/β^0^‐thalassemia with the initiation codon (ATG→ACG) pathogenic variant had an HbA_2_ value of 10% (Figure S2).

**Table 1:** The distribution of β‐thalassemia pathogenic variants within the study population

rs number	HGVS coding	Mutation	Type	Alleles *n* (%)	Mean HbA_2_ (%; 95% CI)^a^
rs33959855	NM_000518.5:c.67G > T	CD22 (GAA➝TAA)	β^0^	55 (66.3%)	4.95 (4.76–5.14)
rs33941849	NM_000518.5:c.2T > C	Initiation codon (ATG➝ACG)	β^0^	20 (24.1%)	5.37 (4.67–6.06)
rs193922563	NM_000518.5:c.93−22_95del	IVS1−3ʹ end del 25 bp	β^0^	6 (7.2%)	4.88 (4.18–5.58)
rs35004220	NM_000518.5:c.93−21G > A	IVS‐I−110 (G➝A)	β^+^	2 (2.4%)	4.30 (NA)

^a^ No significant differences were seen in % HbA_2_ means when comparing β‐thalassemia pathogenic variants to each other (*p* = .25).

Overall, 73 of 310 (24%) and 10 of 116 (9%) of the β‐thalassemia alleles were found among the presumed HbAA and HbSS groups, respectively (Table 2). Within the former, 70 of 73 (95.9%) of the carriers were children with an HbA_2_ of ≥ 4.0%, while only three of 73 (4.1%) had values of 3.5%–3.9%, and none had HbA_2_ values of <3.5%. A similar trend was observed among the HbS‐only group, in which nine of 10 (90%) β^0^‐thalassemia carriers had an HbA_2_ value of ≥4.0% (Table 2). We found no β‐thalassemia alleles among any of the participants with an HPLC pattern suggestive of HbAS. We only identified two participants within this group in whom the concentration of HbS exceeded that of HbA in which the proportions of HbA, HbS, HbF, and HbA_2_ were (1) 21%, 26%, 51%, 2% and (2) 34%, 36%, 27%, 3%, respectively. No β‐globin mutations were identified in either of these participants.

**Table 2:** The prevalence of β‐thalassemia by HbA_2_ categories

HbA_2_ category	HbAA			HbSS			HbAS*		
	Observed β‐thalassemia alleles (*n*/*N* %)	Alleles in total population (P)	Estimated β‐thalassemia alleles within the total population	Observed β‐thalassemia alleles (*n*/*N*) (%)	Alleles in total population (P)	Estimated β‐thalassemia alleles within the total population	Observed β‐thalassemia alleles (*n*/*N*) (%)	Alleles in total population (P)	Estimated β‐thalassemia alleles within the total population
HbA_2_ (< 3.5%)	0/214 (0)	25,382	0	0/102 (0)	106	0	0/198 (0)	1,066	0
HbA_2_ (3.5%–3.9%)	3/218 (1.4%)	584	8	1/40 (2.5%)	50	1	0/202 (0)	1960	0
HbA_2_ (≥4.0%)	70/188 (37.2%)	204	76	9/90 (10%)	96	10	0/208 (0)	1706	0
**Total**			84			11			

Note

The estimated numbers of β‐thalassemia alleles within each subgroup were calculated from the fractions within each Hb type and HbA_2_ category, multiplied by the number of alleles within the same subgroups in the whole population. * No β^+^‐thalassemia alleles were identified in any members of this group, including the two infants in whom the proportion of HbS exceeded that of HbA.

By extrapolation from the β‐thalassemia allele frequencies in each of the HbA_2_ categories, we estimated the allele frequency among members of the Kilifi Genetic Birth Cohort Study overall at 95 of 31,154 (0.3%; Table 2). Assuming Hardy–Weinberg equilibrium, this equates to an approximate birth prevalence for β‐thalassemia heterozygotes and homozygotes of six of 1,000 and one of 100,000, respectively. Given that, on the basis of our HPLC data, the allele frequency for the β^s^ pathogenic variant was approximately 8%, we estimate that the birth prevalence for HbSS within this population approximates to one of 100 and that for HbS/β‐thalassemia to one of 1,000, meaning that approximately 10% of all cases of sickle cell disease within our study population are due to HbS/β^0^‐thalassemia.

We examined the origin of the β‐thalassemia chromosomes we identified by constructing a haplotype map using three common variants: rs12788013, rs1609812, and rs713040. These existed tightly in one block (Dʹ > 0.95) and were at overall minor allele frequencies of 10%, 13%, and 14%, respectively. A total of six haplotypes were identified across this LD block (Table 3 and Figure S1). The CCT haplotype was more common in those with the CD22 (GAA➝TAA) pathogenic variant, occurring at a frequency of 49%. Conversely, the GTC haplotype was more frequent in those without a β‐thalassemia pathogenic variant and in those with other β‐thalassemia pathogenic variants, occurring at the following frequencies: (i) no pathogenic variant 88.9%; (ii) initiation codon (ATG➝ACG) 100%, IVS1‐3ʹ end del 25bp 91.7%; and (iii) IVS‐I‐110 (G→A) 100% (Table 3). On investigating the origin of participants, 47 of 55 (85%) of infants with the CD22 (GAA➝TAA) pathogenic variant were from the Chonyi ethnolinguistic group while all but one (19/20; 95%) of those with the initiation codon (ATG➝ACG) were of non‐Chonyi origin (Figure S3).

**Table 3:** The haplotype distribution by β‐thalassemia pathogenic variants in Kilifi

Haplotype	No β‐thalassemia (*N* = 279)	CD22 (GAA➝TAA) (*N* = 55)	Initiation codon (ATG➝ACG) (*N* = 20)	IVS1−3ʹ end del 25bp (*N* = 6)	IVS‐I−110 (G➝A) (*N* = 2)
GTC	88.9	45.5	100	91.7	100
GCT	3.8	2.8	0	0	0
CCT	3.0	49.1	0	0	0
GCC	1.1	0	0	0	0
GTT	1.8	1.8	0	8.3	0
CTT	1.1	0.9	0	0	0

Note

Haplotypes were constructed from three tagging SNPs: rs12788013, rs1609812, and rs713040. Figures represent column percentages.

Finally, we used the estimated allele numbers from within the HbAA subgroup to calculate the sensitivity, specificity, and positive‐ (PPV) and negative predictive values (NPV) for the diagnosis of heterozygous β‐thalassemia on the basis of our various HbA_2_ cutoffs. A threshold of ≥ 3.5% gave values of 100%, 98%, 21%, and 100%, respectively, while one of ≥ 4% was associated with values of 91%, 100%, 75%, and 100%, respectively (Table 4).When all participants were included in this analysis, including those with presumed HbAS and presumed HbSS, specificity and PPV estimates for an HbA_2_ threshold of ≥ 3.5% dropped substantially to 86% and 4%, respectively, and those for a threshold of ≥ 4% fell to 94% and 9%, respectively (Table S1).

**Table 4:** The diagnostic accuracy of HbA_2_ values in the prediction of β‐thalassemia within the subgroup of participants with HPLC patterns consistent with HbAA

β‐thalassemia	HbA_2_ < 3.5%	HbA_2_ ≥ 3.5%	HbA_2_ ≥ 4%
Present^⨕^	0	84	76
Absent^⨕^	12,691	310	26
Sensitivity (%; 95% CI)	*N*/A	100.0 (94.5–100.0)	90.5 (81.6–95.5)
Specificity (%; 95% CI)	*N*/A	97.6 (97.3–97.9)	99.8 (99.7–99.9)
PPV (%; 95% CI)	*N*/A	21.3 (17.4–25.8)	74.5 (64.7–82.4)
NPV (%; 95% CI)	*N*/A	100.0 (99.9– 100)	100.0 (99.9–100.0)

Note

*N*/A not applicable – no thalassemia alleles detected within this group. ^⨕^The estimated numbers of β‐thalassemia carriers within each subgroup were calculated from the fractions within each Hb type and HbA_2_ category, multiplied by the number of participants within the equivalent subgroup within the whole population.

## 3 DISCUSSION

With the exception of North Africa, where both the prevalence and causal pathogenic variants have been well‐described previously (Agouti, Badens, Abouyoub, Levy, & Bennani, 2008; Habib & Book, 1982; Hamamy & Al‐Allawi, 2013), it is generally thought that β‐thalassemia is rare throughout most of the rest of the continent. In the present study, we screened members of the Kilifi Genetic Birth Cohort Study, recruited on the coast of Kenya, for β‐thalassemia. We detected four different β‐thalassemia pathogenic variants which together affected approximately 0.6% of all study participants.

In a recent report, we showed that HbA_2_ values were consistent with heterozygous β‐thalassemia in a total of 0.8% of Kilifi Genetic Birth Cohort members with an HPLC pattern suggestive of HbAA (Macharia et al., 2019). In the current study, we focused on a number of subgroups within this cohort which we selected on the basis of HbA_2_ value ranges that reflected their relative likelihood of being carriers for β‐thalassemia. Among HbAA participants, we found that an HbA_2_ value of ≥ 4% was associated with a sensitivity of 91% and a PPV of 75% for β‐thalassemia heterozygosity. Nevertheless, despite this high sensitivity, in keeping with previous reports (Gasperini et al., 1993), we found no β‐thalassemia alleles in a quarter of this subgroup. This could be explained by the presence of rare pathogenic variants in the δ‐globin gene or by other causes of elevated HbA_2_, which include megaloblastic anemia, HIV infections, and hypothyroidism (Weatherall & Clegg, 2002), none of which we investigated in the current study.

When considering the entire population, including those with HPLC phenotypes consistent with either HbAS or HbSS, the use of an HbA_2_ level of ≥ 4% resulted in a considerably higher false positivity rate, reflected in a PPV of only 9%. This is almost certainly explained by the presence of glycated HbS and adducts associated with HbS, which have been shown to co‐elute with HbA_2_ and to result in artifactually raised HbA_2_ values when using the BioRad Variant system (Macharia et al., 2019; Suh, Krauss, & Bures, 1996). For this reason, we sequenced all individuals with an HPLC pattern suggestive of HbSS, in whom we found that 10% were compound heterozygotes for both HbS and β^0^‐thalassemia. When these subjects were typed for the rs334 pathogenic variant by PCR, the results were indicative of HbAS, emphasizing the need for confirmatory testing of suspected SCD by more than one diagnostic method. As anticipated, we found no occurrences of β‐thalassemia among those participants who displayed an HPLC pattern that included both HbA and HbS. As β‐globin production is abolished on chromosomes carrying β^0^‐thalassemia alleles, the only β‐globin that would be produced by a β^0^‐thalassemia carrier would be encoded by the complimentary chromosome and, as a result, only one form of β‐globin (either HbA or HbS) would be visible on HPLC. While the same is not true for β^+^‐thalassemia, where a variable amount of residual of β‐globin would be produced, only two children displayed HPLC patterns in which the quantity of HbS exceeded that of HbA. In both these cases this difference was only marginal and was in the presence of high levels of HbF, probably reflecting the accuracy limits of the HPLC method.

To date, more than 300 β‐thalassemia pathogenic variants have been identified worldwide (Kountouris et al., 2014), although approximately 90% of cases are caused by only 40 (Thein, 2018). The majority are point mutations or small deletions or insertions that affect *HBB* function at either the transcriptional, post‐transcriptional or translational stages (De Sanctis et al., 2017; Thein, 2018; Weatherall & Clegg, 2002). Pathogenic variants differ by geographical region with only one or two accounting for > 50% of cases within any given region (Weatherall & Clegg, 2002). In North Africa, for example, IVS110G→A is the most common pathogenic variant, which together with CD39 C→T, IVS1‐1 G→A, and IVS1‐6 T→C is responsible for over 60% of cases (Bennani et al., 1993; Douzi et al.., 2015; Elmezayen, Kotb, Sadek, & Abdalla, 2015; Weatherall & Clegg, 2002). The cluster of pathogenic variants defining β‐thalassemia in Kilifi, observed through this study, is dominated by two β^0^‐thalassemia variants, CD22 (GAA→TAA; 66.3%) and initiation codon (ATG→ACG; 24.1%), while the remaining cases were explained by one additional β^0^‐(IVS1‐3ʹ end del 25bp) and a single β^+^‐thalassemia variant [IVS‐I‐110 (G→A)]. Most are rare in other populations, for example, with the exception of our own previous study (McGann et al., 2018), to the best of our knowledge the main pathogenic variant we found (CD22 (GAA→TAA)) has only previously been reported in one individual from the Reunion Republic (Ghanem et al., 1992) in whom the clinical outcome was not described. Similarly, to date, ATG→ACG has only been reported in three members of one family from Yugoslavia (Wildmann et al., 1993), two family members of Swiss origin (Beris, Darbellay, Speiser, Kirchner, & Miescher, 1993), and two family members of Russian origin (Molchanova, 1998). Notably, in all three studies it was reported that HbA_2_ levels were higher than those commonly seen in other β‐thalassemia pathogenic variants. In this study, we identified 18 β‐thalassemia carriers and two HbS/β^0^‐thalassemia subjects with this variant in whom HbA_2_ levels were also higher than in carriers of other variants, although this observation did not reach statistically significance. Finally, the third most common pathogenic variant observed in this study was IVS1‐3ʹ end del 25bp. First identified by Orkin and colleagues (Orkin et al., 1983) in a patient of Indian origin, this pathogenic variant has since been found to be common in a number of Middle Eastern populations. The highest frequencies have been reported in Bahrain, where in one study it accounted for 36% of all β‐thalassemia alleles (Jassim, Al‐Arrayed, Al‐Mukharraq, Merghoub, & Krishnamoorthy, 2000). However, in other Middle Eastern countries the frequencies of this pathogenic variant are not as high, being 7.3% in Kuwait (Adekile et al., 1994), 9.5% in United Arab Emirates (el‐Kalla & Mathews, 1997), and 12.9% in Saudi Arabia (el‐Hazmi, Al‐Swailem, & Warsy, 1995). In the current study IVS1‐3ʹ end del 25bp explained 6.8% of the β‐thalassemia alleles, and was the cause of HbS/β^0^‐thalassemia in one participant. A single occurrence of the β^+^‐thalassemia pathogenic variant IVS‐I‐110 (G→A), a common variant in North African, European and the Middle Eastern countries (Agouti et al., 2008; Bennani et al., 1993; Douzi et al., 2015; Elmezayen et al., 2015; Weatherall & Clegg, 2002), was identified in our study. Although a number of reports have suggested that the −29 (C→G) pathogenic variant could have originated from sub‐Saharan Africa (Gonzalez‐Redondo et al., 1991; Weatherall & Clegg, 2002), we found no occurrences of this pathogenic variant in our current cohort or in our previous study conducted in Kenya, Uganda, Angola, and the Democratic Republic of Congo (McGann et al., 2018).

HbS/β‐thalassemia results from coinheritance of both a β‐thalassemia and a β^s^ pathogenic variant on contralateral chromosomes. Although the clinical manifestations of HbS/β‐thalassemia are thought to be generally similar to those of HbSS, they can vary depending on the type of β‐thalassemia mutation that is co‐inherited, which in turn varies from one region to another (Steinberg, Forget, Higgs, & Weatherall, 2009). In particular, depending on the amount of β‐globin produced, individuals of the HbS/β^+^‐thalassemia can have a milder form of SCD compared to HbS/β^0^‐thalassemia and HbSS (Jha, Mishra, Verma, Pandey, & Lakkakula, 2018; Serjeant, Sommereux, Stevenson, Mason, & Serjeant, 1979; Yadav et al., 2016). Describing the disease phenotypes that are associated with these mutations is important because it can inform guidelines on the better management of individuals suffering from these conditions. In the current cohort we identified 10 individuals with HbS/β^0^‐thalassemia; a full description of their clinical phenotype is an aim of ongoing work.

In our haplotype analysis, we found that CD22 (GAA→TAA) was strongly linked to the CCT haplotype, which is different from the background haplotype of the general population (GTC).This suggests that this pathogenic variant may have arrived in Kilifi through gene flow or population migration and that it is unlikely to have arisen on the chromosomal background of the current population. By contrast, the remaining β‐thalassemia pathogenic variants were exclusively linked with the native haplotype, suggesting that these pathogenic variants may have occurred de novo within this population. Examination of the ethnic background on which both the different pathogenic variants and haplotypes were found suggests that both relate to specific subpopulations within the cohort as a whole. Further work will be required to investigate the origins of these pathogenic variants in more detail.

On the basis of our current study, we estimate that approximately 0.6% of the Kilifi population carry a β‐thalassemia pathogenic variant, which means that some cases of β‐thalassemia major should be seen. Nevertheless, we have not previously identified a suspected case within our clinical practice, and, to the best of our knowledge, β‐thalassemia major has not been reported from anywhere else in the East Africa region. The most likely explanations for this discrepancy relate to the low predicted birth rate for homozygotes, historically high rates of both all‐cause child mortality and severe acute anemia (Macharia et al., 2019), and the lack of diagnostic facilities. Under these circumstances, it seems likely that most homozygotes will have been dying in early life without coming to medical attention. Whatever the explanation, as both malaria and all‐cause mortality decline, it is likely that affected children will increasingly survive to the point at which their disease will be recognizable and, as such, clinicians within the region should learn more about β‐thalassemia including appropriate methods for its diagnosis and treatment.

Our study has several weaknesses of which the most important were the age range of the children studied and the method by which blood was taken. Switching to adult patterns of hemoglobin production may well have been incomplete, particularly in the youngest subgroup, making the classification of HbA_2_ values potentially misleading. In addition, the volumes of blood collected did not allow us to refine our screening strategy to take account of data from complete blood counts or to investigate children for alternative causes of raised HbA_2_ values, which include megaloblastic anemia, HIV infections, and hypothyroidism (Weatherall & Clegg, 2002). In contrast, these weaknesses do not detract from the central message of our study which is that β‐thalassemia is present in a region in which it was previously not known to occur. While deficiencies in the study design might have led us to miss some cases, our study still provides a minimum estimate of the true prevalence of β‐thalassemia within the study population.

In conclusion, we have described the prevalence and spectrum of β‐thalassemia in a cohort of children on the coast of Kenya, a region in which this condition has not been previously recognized. In addition, we show the sensitivity of various HbA_2_ threshold values as a method for β‐thalassemia screening in this population and speculate on the possible origin of the pathogenic variants we have identified. Our study has implications for the clinical care of children from the East Africa region.

## 4 METHODS

### 4.1 Study design

The study was conducted among members of the Kilifi Genetic Birth Cohort Study that was designed to investigate the impact of host genetic factors on a range of common child health outcomes. The methods for recruitment to this study have been described in detail previously (Macharia et al., 2019; Williams et al., 2009). Briefly, infants 3–12 months of age who were born in the area served by the Kilifi Health and Demographic Surveillance System (KHDSS) between January 2006 and April 2010 were eligible for inclusion in the study (Scott et al., 2012). Capillary blood samples collected from the heel were used for HPLC analysis and DNA extraction. The method and volumes collected did not allow for the conduct of additional tests, including full blood count analysis. The proportions of specific types of hemoglobin were assayed at enrolment on a Variant Classic^TM^ HPLC analyser using the β‐thalassemia Short Program (BioRad,Hercules, CA, USA). HbA_2_ values were within the β‐thalassemia range in a small proportion of recruited children (Macharia et al., 2019), a group that forms the primary focus for the current study.

### 4.2 Study participants

First, among participants with an HPLC pattern suggestive of HbAA (a pattern showing the presence of HbA and the absence of any abnormal variants), we sequenced subjects selected on the basis of their recruitment HbA_2_ values as follows. We sequenced all children with HbA_2_ values of ≥ 4.0%, a group in whom we expected to find a high proportion of β‐thalassemia carriers on the basis of published reports (Van Delft et al., 2009; Mosca, Paleari, Ivaldi, Galanello, & Giordano, 2009). We also sequenced a random sample of 110 participants whose values were between 3.5% and 3.9%, a cutoff associated with a high sensitivity but low specificity for β‐thalassemia (Van Delft et al., 2009; Mosca et al., 2009). Finally, we sequenced a random sample of 114 participants whose values of <3.5%, in whom β‐thalassemia should be rare. Next, because the interpretation of HbA_2_ values in relation to the diagnosis of β‐thalassemia is less clear in the presence of HbS (Suh et al., 1996), we sequenced all participants with an HPLC pattern suggestive of HbSS and a subset of participants with HPLC patterns suggestive of HbAS as follows: (1) a random sample of ~100 participants with each of the HbA_2_ cutoffs outlined for HbAA subjects above; (2) all participants with HPLC patterns that showed both HbA and HbS but in whom the concentration of HbS exceeded that of HbA, a pattern consistent with a diagnosis of HbS/β^+^‐thalassemia (Weatherall & Clegg, 2002).

### 4.3 DNA extraction and *HBB* sequencing

Genomic DNA was extracted for all members of the Kilifi Genetic Birth Cohort Study from capillary blood samples collected into EDTA within 7 days of their recruitment using an ABI PRISM 6100 Nucleic AcidPrepStation™ (Applied Biosystems). For the current study, β‐globin gene sequencing was conducted on selected participants using samples that had been stored at −80°C. We sequenced amplicons derived by PCR using *HBB* primers using an ABI 3730xl sequencer and the BigDye Terminators cycle Sequencing Kit (Applied Biosystems), as described in detail previously (Clark & Thein, 2004). In brief, our sequencing method covered a 1.8kb region that included the 5ʹ promoter, 5ʹ and 3ʹ untranslated regions, exons 1–3, and the intervening sequence I and II regions flanking exons 2 and 3.

### 4.4 Statistical analysis

Differences in mean HbA_2_ percentages between different β‐thalassemia pathogenic variants were analyzed using one‐way ANOVA. *p* values of < 0.05 were considered statistically significant. All statistical analyses were performed using the R Foundation for Statistical Computing Platform Version 3.1.1 (The, 2017). Linkage disequilibrium analysis and the construction of haplotypes were carried out using Haploview Version 4.2 (Barrett, Fry, Maller, & Daly, 2005).

## 5 AUTHOR CONTRIBUTORS

AWM, SU, CMN, REW, and TNW designed the study and conducted the literature review. GM, collected the clinical data while AWM, JM, MT, GN, and EN assisted with sample preparation and analysis. AWM analyzed data and all the authors helped to interpret the data. AWM, TNW, and SU wrote the first draft of the paper. All the authors contributed to editing the final version.

## CONFLICT OF INTEREST

None of the authors have any conflict of interest to declare.

## ETHICS

Individual written informed consent was provided by the parents of all study participants. Ethical approval for the study was granted by the Kenya Medical Research Institute/National Ethical Review Committee in Nairobi, Kenya (reference number SCC 1058).

## Supporting information

Supplementary Material

## Data Availability

The datasets generated and analyzed during the current study are not publicly available because specific permission for public deposition was not obtained at the time of informed consent but are available from the corresponding author on reasonable request.
