# Supplementary material for: β‐Thalassemia pathogenic variants in a cohort of children from the East African coast
Source: Mol Genet Genomic Med. 2020 May 11;8(7):e1294. doi: 10.1002/mgg3.1294 (PMC7336762; doi:10.1002/mgg3.1294)

Table S1. Diagnostic accuracy of HbA_2 in predicting β–thalassemia within the entire population, including those with HbAS and HbSS.

**β–thalassemia**	**HbA_2 <3.5%**	**HbA_2 ≥3.5%**	**HbA_2 ≥4%**
Present[§]	0	95	86
Absent[§]	13277	2205	917
Sensitivity (%; 95% CI)	N/A	100.0 (95.2-100.0)	90.5 (82.3-95.3)
Specificity (%; 95% CI)	N/A	85.8 (85.2-86.3)	94.0 (93.7-94.4)
PPV (%; 95% CI)	N/A	4.1 (3.4-5.0)	8.6 (7.0-10.5)
NPV (%; 95% CI)	N/A	100 (99.9-100.0)	99.9 (99.9-100.0)

N/A Not applicable – no alleles detected within this group. [§]The estimated numbers of β–thalassemia carriers within each sub-group were calculated from the fractions within each Hb type and HbA_2 category, multiplied by the number of participants within the equivalent sub-group within the whole population.

Figure S1. Haplotype block and linkage disequilibrium (LD) of 3 tag SNPs rs12788013, rs1609812 and rs713040. Pairwise correlation values (D`) are shown as numbers inside the squares. The red squares represent high level of LD between two SNPs.

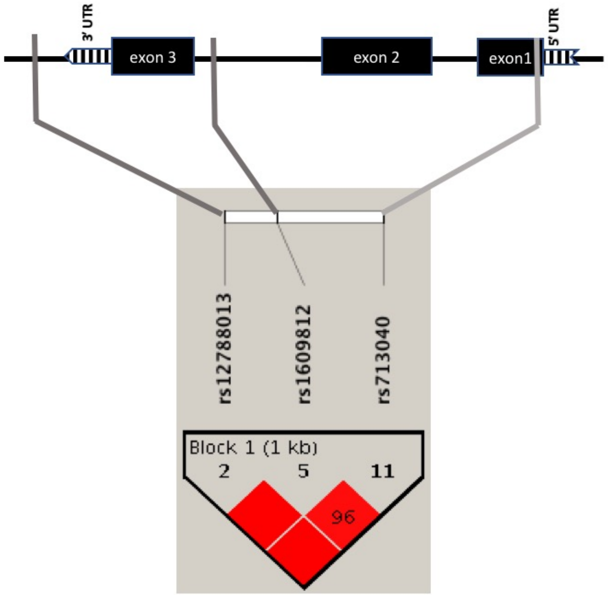

Figure S2. HbA_2 levels classified by HPLC haemoglobin phenotype and presence or absence of β-thalassemia mutations.

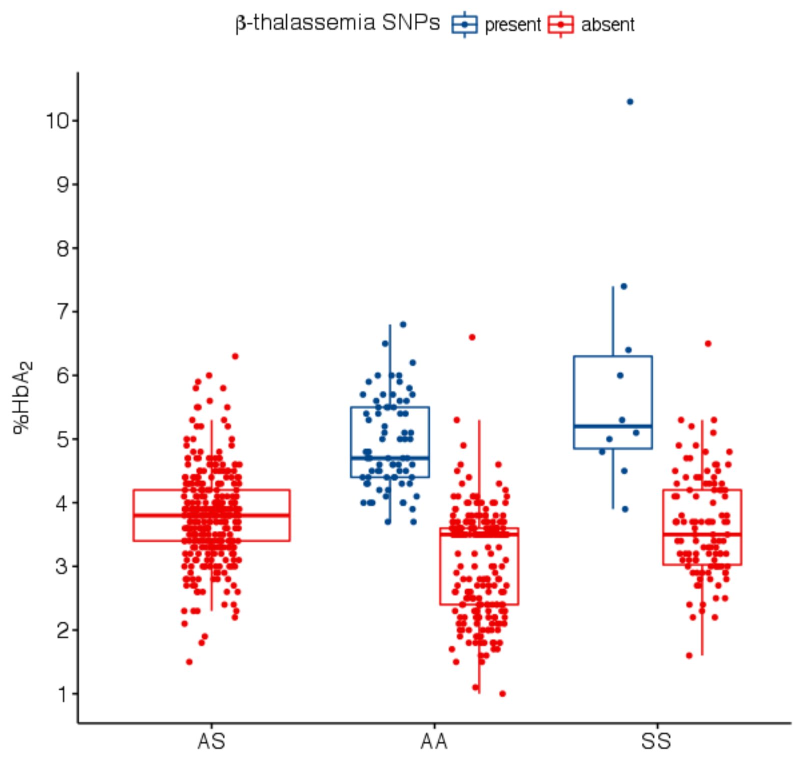

Figure S3. Distribution of β-thalassemia mutations using ethnolinguistic background.

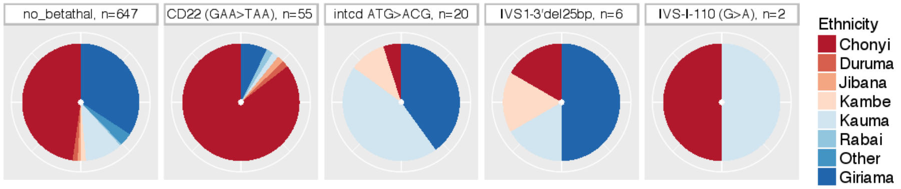

Supplement: Supplementary file 1 — Supplementary Material [file MGG3-8-e1294-s001.pdf]
